# Effect of Sn Grain Orientation on Reliability Issues of Sn-Rich Solder Joints

**DOI:** 10.3390/ma15145086

**Published:** 2022-07-21

**Authors:** Yu-An Shen, John A. Wu

**Affiliations:** 1 Department of Materials Science and Engineering, Feng Chia University, Taichung 407, Taiwan; 2 School of Materials Engineering, Purdue University, West Lafayette, IN 47907, USA; wu1696@purdue.edu

**Keywords:** lead-free solder, Sn grain orientation, electromigration, thermomigration, thermomechanical fatigue, Sn orientation control

## Abstract

Sn-rich solder joints in three-dimensional integrated circuits and their reliability issues, such as the electromigration (EM), thermomigration (TM), and thermomechanical fatigue (TMF), have drawn attention related to their use in electronic packaging. The Sn grain orientation is recognized as playing an important role in reliability issues due to its anisotropic diffusivity, mechanical properties, and coefficient of thermal expansion. This study reviews the effects of the Sn grain orientation on the EM, TM, and TMF in Sn-rich solder joints. The findings indicate that in spite of the failure modes dominated by the Sn grain orientation, the size and shape of the solder joint, as well as the Sn microstructures, such as the cycling twining boundary (CTB), single crystals, and misorientations of the Sn grain boundary, should be considered in more detail. In addition, we show that two methods, involving a strong magnetic field and seed crystal layers, can control the Sn grain orientations during the solidification of Sn-rich solder joints.

## 1. Introduction

Lead-free solder has been widely used in electronic packaging since the restriction of the inclusion of lead in consumer electronics [1,2,3]. Lead-free solders are Sn-based alloys with various kinds of element additions, including Ag, Cu, Ni, Bi, Zn, Ti, In, and graphene [4,5,6,7,8,9,10]. Such alloys possess a wide range of melting points, electrical and mechanical properties, microstructures, and wetting behaviors for various applications in electronic devices [11,12,13,14,15]. Among them, Sn-rich solder, which contains considerable amounts of Sn in the solder matrixes, is widely adopted in solder joints that are assembled via under-bump metallization (UBM), using a solder alloy and intermetallic compound (IMC) at the interface between the solder and the UBM method via a reflow process at a suitable temperature [16,17,18]. The reliability issues of Sn-rich solder joints have drawn substantial concerns, the first of which is the EM reliability during a consistent electron flow with a critical current density in Sn-rich solder joints [19,20,21], inducing UBM dissolution, rapid IMC growth, void formation, and severe Joule heating [22,23,24,25]. Then, the Joule heating induces a temperature gradient in the solder joint, known as the TM [22,26,27,28]. Additionally, during multiple cycles of increases and decreases in the internal temperature, i.e., thermal cycling tests, the TMF is another important reliability issue because of the CTE mismatches between the Sn grains, Si chip and polymer substrate, Sn-rich solder and Si chip, and Sn-rich solder and polymer substrate [29,30,31]. However, the properties of Sn-rich solders are dominated by β-Sn crystals, and the effects of their properties on the EM, TM, and TMF of Sn-rich solder joints are worth investigating in detail.

Sn possesses a body-centered tetragonal structure (a-axis: 0.583 Å, c-axis: 0.318 Å at 25 °C) [32], inducing anisotropic diffusion and thermal, mechanical, and electrical properties [33,34,35,36,37,38,39]. The effects of these anisotropic properties on the EM, TM, and TMF reliability levels of solder joints are critical issues that have been deeply studied by many researchers. Moreover, due to the miniaturization and high performance of electronic devices, three-dimensional integrated circuits (3DICs) have become very popular and critical for the next generation of electronic packaging [40,41,42]. The use of a microbump is particularly important to connect the through-silicon-via (TSV) and chip in 3DICs. However, with the reduction in solder volume, the Sn-rich solder in the microbumps may contain few Sn grains or even a single-crystal-like structure. The effects of the Sn grain orientation on these issues are more significant than in flip–chip solder joints because of the dominant grains in the microbumps. These issues are very important, and this review paper aims to figure out the effects of the Sn orientation on the mechanical, electrical, and thermal behaviors of solder joints. First, we determine which Sn orientations are suitable for different electronic packaging designs. Consequently, this paper discusses the effects of the Sn grain orientation on the EM, TM, and TMF in Sn-rich solder joints. The methods used to control the Sn grain orientation are also discussed in this study.

## 2. Effect of Sn Grain Orientation on Electromigration

**Figure 1:**
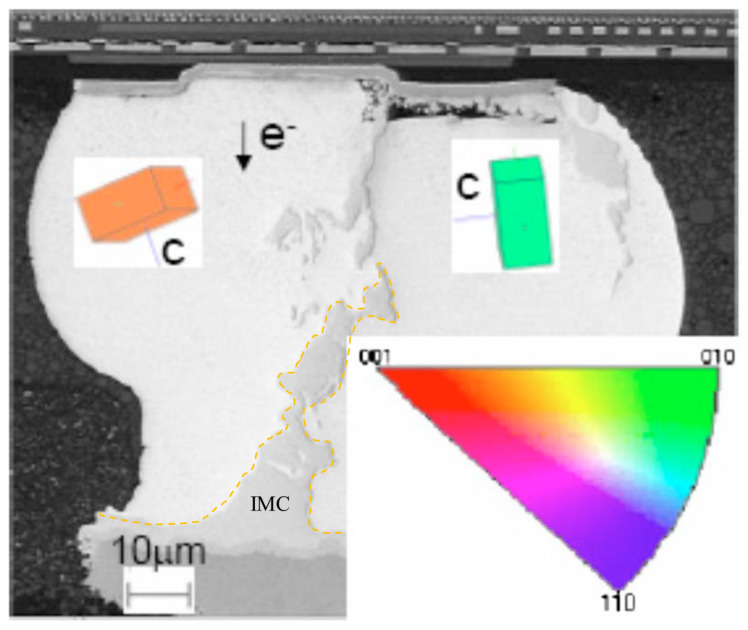
SEM image of a solder joint within two Sn grains on the c-axis and a-axis closely parallel to the electron flow (the arrow points in the flow direction of the electrons) Reprinted with permission from ref. [49] Copyright 2008 AIP Publishing. Rapid IMC growth in the grain of the c-axis closely parallel to the electron flow (yellow dashed line).

Electromigration is the phenomenon whereby atomic migration is caused by the momentum transfer between electrons and diffusing specimens under an electric current [43,44,45,46,47,48]. The atomic flux diffusing during electromigration can be expressed as [19]:

(1) $$J_{EM}=C\frac{D}{KT}Z^{*}eE=C\frac{D}{KT}Z^{*}e\rho j$$

where *C* is the atomic concentration, *D* is the diffusivity, Z* is the effective charge number, *K* is Boltzmann constant, *T* is the absolute temperature, *e* is the electron charge, *E* is the electron field, *ρ* is the resistivity, and *j* is the current density. Because interstitial diffusion dominates the electromigration behavior of Sn-rich solder joints, as the Sn self-diffusion rate is very low [33], the considerable atoms of UBMs would migrate in Sn solder from the cathode to the anode. Meanwhile, the anisotropic diffusion of Sn grains induces different failure modes due to the different atomic fluxes. Table 1 summarizes the diffusivity levels of Cu and Ni along the c-axis and a-axis during EM in Sn at 120 °C. The diffusivity rate of Cu along the c-axis of Sn is approximately 61 times larger than that along the a-axis. For Ni, the diffusivity rate along the c-axis is approximately 70,000 times larger than that along the a-axis, being much greater. Therefore, the effect of the Sn grain orientation on EM failure in Sn solder joints with Ni UBMs under a current density of 7.7 × 10^3^ A/cm^2^ was first observed by Lu et al. [49]. Since then, substantial studies on this effect have been reported [50,51,52,53,54,55]. In the studies, the extra-fast UBM dissolution of Cu/Ni at the cathode occurs as the electron flow is closely parallel to the c-axis, which possesses a high diffusion rate. However, when the electron flow is vertical to the c-axis, i.e., closely parallel to the a-axis of Sn, void formations can be observed instead of the depletion of UBM. The UBM dissolutions and void formations rapidly increase the resistivity of the solder joints, which is a critical reliability issue. On the other hand, on the anode side, considerable IMC growth can be observed when the electron flow is parallel to the c-axis of Sn, as shown in Figure 1. In many studies, in addition to increasing the resistivity, the growth of brittle IMC induced poor mechanical reliability in Sn-rich solder joints [56,57,58,59,60]. Therefore, the mechanism of the effect of the Sn grain orientation on the electromigration is very important.

**Figure 2:**
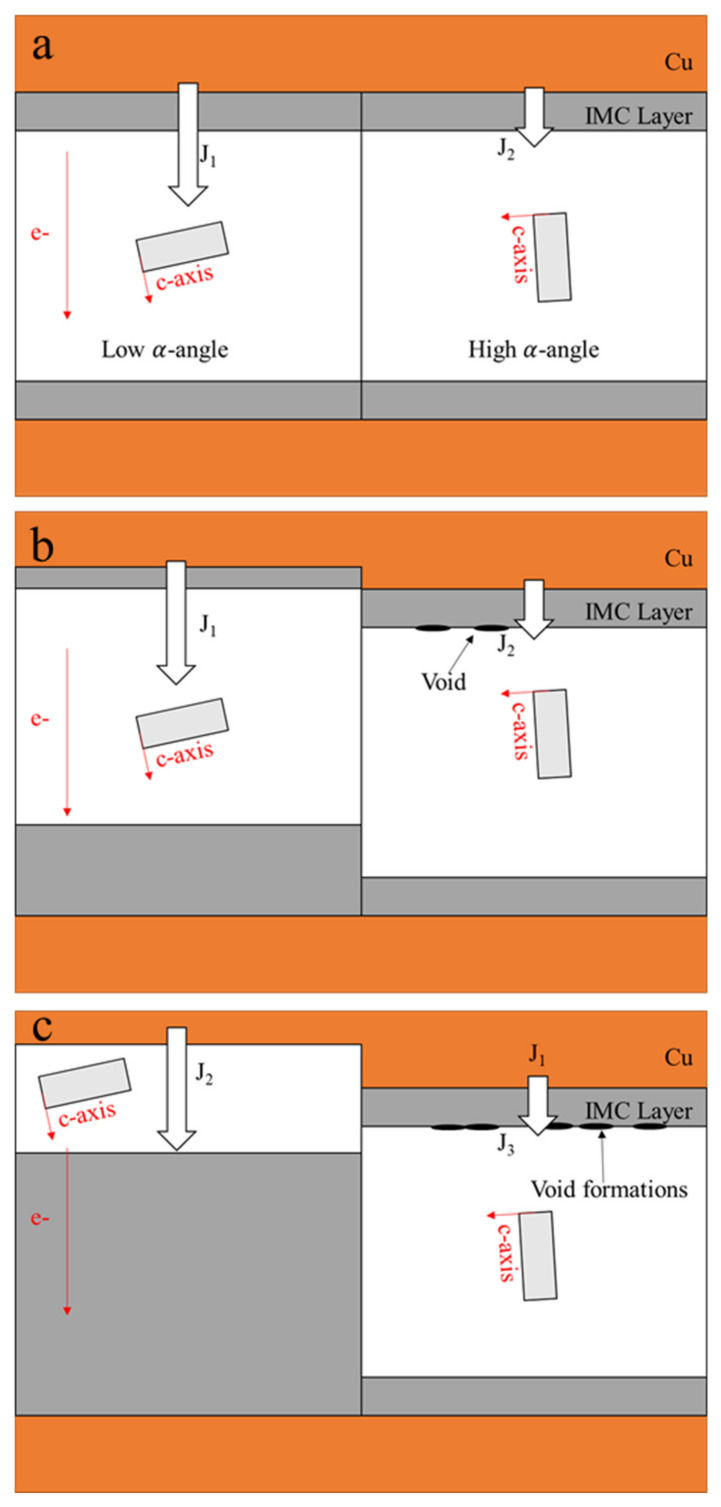
A schematic of the different failure modes induced by the grain orientation in solder joints: (**a**) initial stage; (**b**) intermediate duration; (**c**) terminal stage.

**Figure 3:**
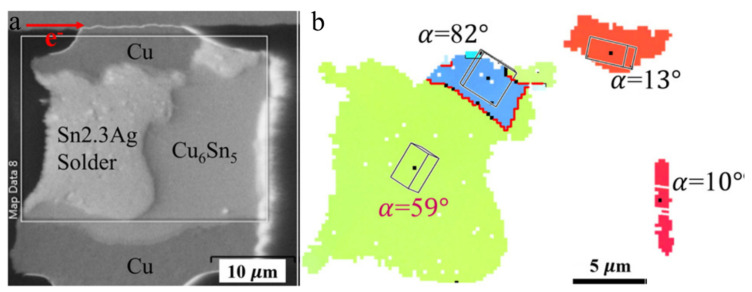
(**a**) A solder microbump with rapid IMC formation at the anode side of the low α-angle grain after EM and (**b**) its EBSD orientation map Reprinted with permission from ref. [61]. Copyright 2016 Elsevier.

In this section, the α-angle is defined as the angle between the c-axis of Sn and the electron flow. The diffusivity of Sn grains in Cu can be expressed as [34]:

(2) $$D_{grain}=D_{c,Cu}\;{cos}^{2}\alpha +D_{a,Cu}\;{sin}^{2}\alpha \;{\mathrm{cm}}^{2}/s$$

where the diffusivity levels along the c-axis (*D_c,Cu_*) and a-axis (*D_a,Cu_*) can be expressed as [33]:

(3) $$D_{c,Cu}=1\times 10^{-3}exp\left[-\frac{4000}{RT}\right]\;{\mathrm{cm}}^{2}/s.$$

(4) $$D_{a,Cu}=2.4\times 10^{-3}exp\left[-\frac{7900}{RT}\right]\;{\mathrm{cm}}^{2}/s$$

where *R* is the universal gas constant and *T* is the absolute temperature. The temperatures directly affect the diffusivity levels, and a higher temperature induces a higher atomic flux during the diffusion, accelerating the occurrence of the IMC growth and UBM dissolution. Hence, when the Sn solder has a low α-angle grain, a considerable amount of atoms from the dissolution of UBMs at the cathode diffuse to the anode. Conversely, instead of fast interstitial diffusion along a low α-angle grain, the Sn self-diffusion dominates the EM, inducing void formations and a little UBM dissolution at the cathode. Figure 2a shows a Cu/Sn-rich solder–Cu joint with high and low α-angle grains of Sn. J_1_ and J_2_ are the Cu fluxes via a low and high α-angle grain, respectively. Owing to J_1_ >> J_2_, the IMC decomposition caused by the fast interstitial diffusion along the low α-angle grain is rapid at the cathode, while void formation occurs in the high α-angle grain, as shown in Figure 2b. With the passing of time (Figure 2c), in the low α-angle grain, serious UBM dissolution at the cathode after the complete decomposition of as-bonded IMC and the substantial IMC growth at the anode are observed; conversely, in the high α-angle grain, there is no significant change in IMC thickness due to the very low J_2_ and the greater number of void formations at the cathode. Moreover, in Figure 3 [61], after an EM test of 65 h under a current density of 4 × 10^4^ A/cm^2^ at 165 °C, the as-bonded Cu_6_Sn_5_ IMC in the solder microbump decomposed at the cathode of an Sn grain of 59°. Considerable IMC growth at the anode and serious UBM dissolution at the cathode were clearly observed in an Sn grain of 13°. Their diffusivity levels were 5 times different according to Equation (2). The effect of the Sn grain orientation is more significant in small-scale solder joints.

**Figure 4:**
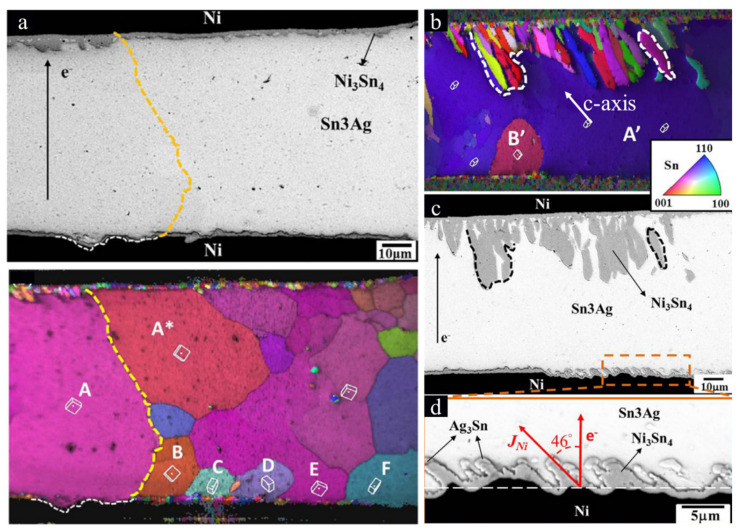
(**a**) A line-type solder joint with high α-angle grains after EM and its EBSD orientation map. The arrow of e^-^ points in the flow direction of electron. (**b**) An EBSD orientation map of (**c**) a line-type solder joint after EM for 400 h. (**d**) The IMC dissolution in the origin frame of (**c**) (the arrow of e^-^ and the arrow of J_Ni_ point in the flow direction of electron and the Ni flux, respectively). Reprinted with permission from ref. [69]. Copyright 2014 Elsevier.

**Figure 5:**
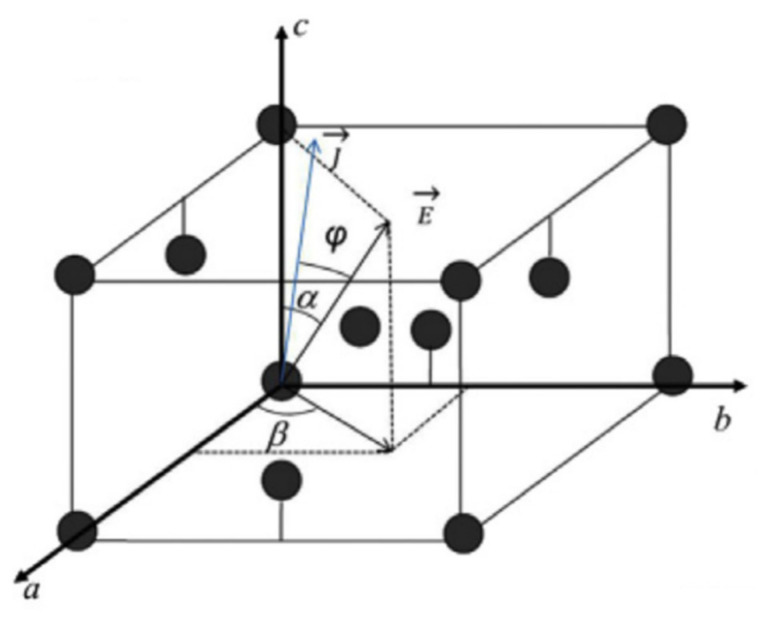
A schematic of the vectors of the electric field and EM flux, as well as Sn a-, b-, and c-axes. Reprinted with permission from ref. [71]. Copyright 2013 AIP Publishing.

However, the void formations and UBM dissolutions at the cathode of the high α-angle grain might be influenced by current crowding during electromigration [62,63,64,65]. Line-type solder joints were fabricated to avoid the complication of current crowding, providing a purer surrounding to observe the effect of the Sn grain orientation on the electromigration in Sn-rich solder joints [66,67,68,69]. UBM dissolution dominated by the Sn grain orientation was observed in the line-type solder joint, without the current crowding effect [69]. As current crowding played no role in the line-type solder joints, there were no obvious void formations at the cathode of the high α-angle grain after EM at a 10^4^ A cm^−2^ current density for 400 h, as shown in Figure 4a. In Figure 4b, an EBSD orientation map is shown for the c-axis direction in the line-type solder joint from Figure 4c. Interestingly, the formation of the Sn-Ni IMC at the anode (Figure 4c) and the dissolution of the Ni substrate at the cathode (Figure 4d) occur along with the angles of the c-axis of Sn grains. The detailed mechanism behind this has been proven in [70]. Figure 5 shows the components of the electron field ($\vec{E}$), Sn unit cell, and the atomic flux ($\vec{J}$) during EM. There is an φ-angle between the $\vec{E}$ and $\vec{J}$ and an α-angle between the c-axis and $\vec{E}$. The φ-angle can be calculated by [71]:

(5) $$cos\varphi =\frac{D_{a}sin^{2}\alpha +D_{c}cos^{2}\alpha }{\sqrt{D_{a}^{2}sin^{2}\alpha +D_{c}^{2}cos^{2}\alpha }}$$

where *D_a_* and *D_c_* are the diffusivity levels, respectively, along a-axis and c-axis in different materials. If the α-angles are 28.61° and 58.57°, the φ-angles will be 28.28° and 57.66°, respectively. The angles are nearly identical to each other. Additionally, the atomic flux $\vec{J}$ can be expressed as [71]:

(6) $$\left|\vec{J}\right|=C\frac{D}{KT}Z^{*}eE\sqrt{\left(D_{a}^{2}sin^{2}\alpha +D_{c}^{2}cos^{2}\alpha \right)}$$

The atomic flux along the electron field (*J^E^*) can be expressed as:

(7) $$J^{E}=\left|\vec{J}\right|cos\varphi =C\frac{D}{KT}Z^{*}eE\left(D_{a}sin^{2}\alpha +D_{c}cos^{2}\alpha \right)$$

As mentioned above, when the α-angle is very low, the atomic flux deviation from the c-axis will become largely considerable. Consequently, the atoms of the UBM mainly migrate along the c-axis from the cathode to the anode through the interstitial diffusion because the contribution of $D_{a}{sin}^{2}\alpha$ is low and *D_c_* ≫ *D_a_*. In a high α-angle grain, the *J^E^* is very low because $\left(D_{a}{sin}^{2}\alpha +D_{c}{cos}^{2}\alpha \right)$ is very small, and even $≅D_{a}{sin}^{2}\alpha$ could be neglected in the comparison with a low α-angle grain. Meanwhile, the atomic flux deviation from the c-axis is very limited, and the UBM dissolution and IMC formation seldom occur along the c-axis. Similar results in the solder joints with Cu UBMs were also observed in [72,73,74]. Moreover, in the Sn-Pb solder microbump, when the electromigration test was carried out at −196 °C, the anisotropic migration of the Pb did not occur. Instead, the study found that the Pb migrated in a parallel path. On the contrary, the Pb rapidly migrated along a specific direction (the c-axis of the Sn) during the electromigration at room temperature [75]. The different migration routes of the Pb were due to the different crystal structures of the Sn at the two temperatures. At <13 °C, the Sn type was α-Sn with a face-centered cubic structure [76], so the electric properties of α-Sn were isotropic. At room temperature, the Sn type was β-Sn with a body-centered tetragonal structure, which was anisotropic [77]. The results exhibit the significant effect of the β-Sn grain orientation on the electromigration in solder microbumps. Therefore, the electromigration along the Sn c-axis is clearly explained.

**Figure 6:**
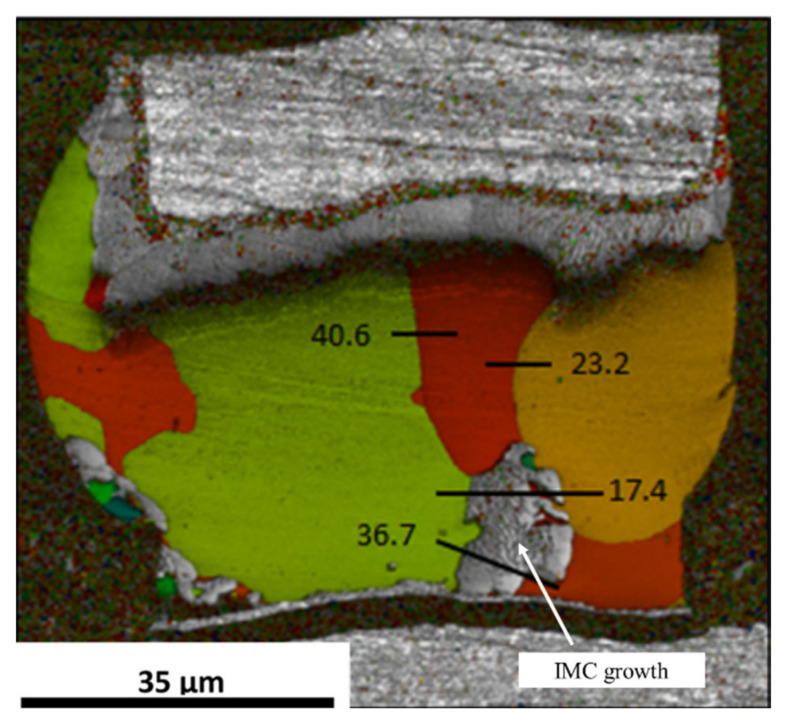
Cu-Sn IMC growth along the grain boundaries in a Sn-0.7Cu solder joint. Reprinted with permission from ref. [78]. Copyright 2014 Springer Nature.

Although the effect of the Sn c-axis on the electromigration is known, Figure 6 shows that the IMC formations occurred along the grain boundaries in Sn-0.7 wt.% Cu solder joints after EM at 165 °C with a ~2.5 × 10^4^ A/cm^2^ current density [78] because the Cu diffusion in Sn along a grain boundary is 10^6^ larger than that along the lattice [79]. This phenomenon would be more significant with the increase in the misorientation angles of the grain boundaries. However, due to the considerable number of Sn cyclic-twin boundaries, which are a type of coherent boundary in β-Sn crystals [80], in Sn-Ag [61] the atoms rarely diffuse along the CTB compared to the boundaries with high misorientation angles. In other words, the effects of grain boundaries on electromigration would be dependent on the type of grain boundary. If there is no CTB in a Sn-rich solder, the UBM dissolutions and IMC formations are influenced by the grain boundary misorientation angles rather than the Sn grain orientations [78]. This is why the EM damages were retarded by the CTB [49,61].

## 3. Effect of Sn Grain Orientation on Thermomigration

Thermomigration, known as the Ludwig–Soret effect, is a type of mass transport caused by momentum transfer between diffusing atoms and electrons under a thermal gradient [27]. Due to the large current densities, the Joule heat can generate a considerable temperature gradient from electron in-flow to out-flow areas in the solder joint, which makes the solder suffer from TM [28,81,82,83]. The atomic flux caused by TM is described as [28]:

(8) $$J_{TM}=-c\frac{D}{kT}\frac{Q^{*}}{T}\frac{dT}{dx}$$

where *C* is the concentration of atoms, *D* is the diffusivity, *k* is Boltzmann’s constant, T is the absolute temperature, Q* is the heat of transport, and *dT/dx* is the temperature gradient. Due to the Cu diffusivity in Sn being four orders larger than Sn self-diffusion, the Cu migration is in result dominant compared to the Sn migration in Sn-rich solder joints during the thermomigration. The Cu migration caused by Sn anisotropic diffusion is an important factor in UBM dissolution and IMC formation in Sn-rich solder joints. However, few studies on the influence of the Sn grain orientation on thermomigration in Sn-rich solder joints are reported.

**Figure 7:**
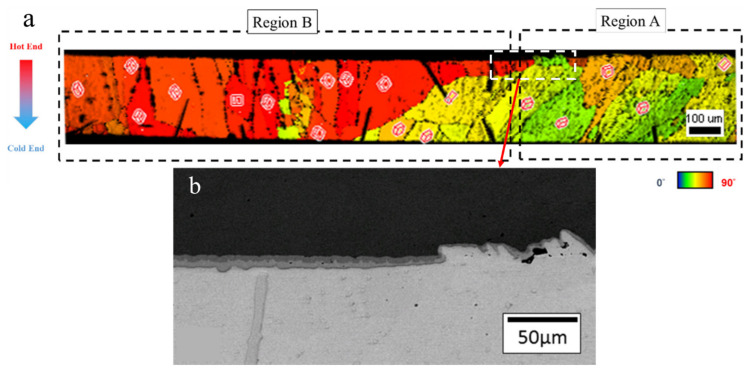
(**a**) The orientation map in a solder joint with a solder height of 282 μm after thermomigration. The Sn c-axes in Region A and B are near parallel and vertical to the direction of the thermal gradient, respectively. The boxes in the picture show the crystalline structure of Sn. (**b**) SEM image of the circled area in the white dashed box of (**a**). Reprinted with permission from ref. [84]. Copyright 2014 Elsevier.

TM degradation depending on the Sn grain orientation was first reported by Hsu et al. [84]. Figure 7a shows the orientation map in a solder joint, with region A, where the Sn c-axis is parallel to the thermogradient, and region B, where the Sn c-axis is vertical to the thermogradient. The solder joint with a solder height of 282 μm is examined between a heat source at 200 °C and a heat sink at 100 °C. Obviously, the dissolution of the IMC and Cu substrate in region A is much more obvious than that in region B at the hot end, as shown in Figure 7b. The atomic flux of Cu along the temperature gradient, *J^TG^*, is expressed as:

(9) $$J^{TG}=-c\frac{D}{kT}\frac{Q^{*}}{T}\left|\nabla \vec{T}\right|\left(D_{a}sin^{2}\alpha +D_{c}cos^{2}\alpha \right)$$

where α is the angle between the c-axis and the direction of the thermogradient. Because the ratio of *Dc*/*Da* is approximately 43 at 150 °C, Equation (9) can be expressed as:

(10) $$J^{TG}=-c\frac{D}{kT}\frac{Q^{*}}{T}\left|\nabla \vec{T}\right|D_{a}\left(1+42cos^{2}\alpha \right)$$

**Figure 8:**
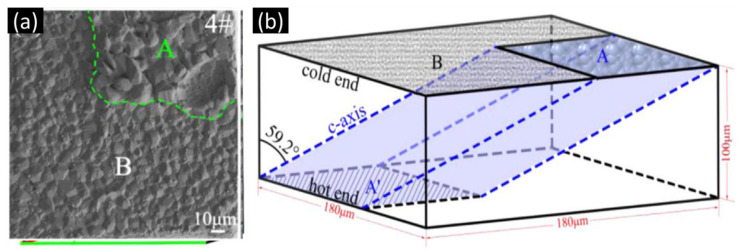
(**a**) SEM image at the planar interface between the solder and Cu at the cold end. The region in the green dash box is the IMC growth area along the Sn c-axis, which corresponds to Region A in (**b**). (**b**) The IMC formation of the uneven regional Cu_6_Sn_5_ IMC at the cold-end interface in regions A and B. A and A’ are the regions for Cu migrations along the Sn c-axis, and the region of B is out of them. Reprinted with permission from ref. [87]. Copyright 2021 Elsevier.

When the c-axis is closely parallel to the direction of the thermogradient (i.e., low α-angle), the $J^{TG}$ is high, inducing serious Cu dissolution at the hot end during thermomigration. Alternatively, at a small $cos^{2}\alpha$ (i.e., high α-angle), the dissolution is small, as shown in region B of Figure 7b. The effect of the Sn grain orientation on the thermomigration has been proven and reported in previous studies [85,86]. Recently, Qiao observed uneven IMC growth at the cold-end interface [87], whereby IMC formations will form two regions, A and B (Figure 8a). The regions of Cu-Sn IMC formations at the cold end were controlled by the angle between the Sn c-axis and the direction of the thermal gradient. According to their calculations, the change in IMC thickness in region A is very consistent with Equation (10) with α = 59°, and Figure 8b shows a schematic of the three-dimensional IMC growth in region A. Similar results are also presented in another article [88]. The findings prove that the Cu diffusion is almost along the Sn c-axis.

**Figure 9:**
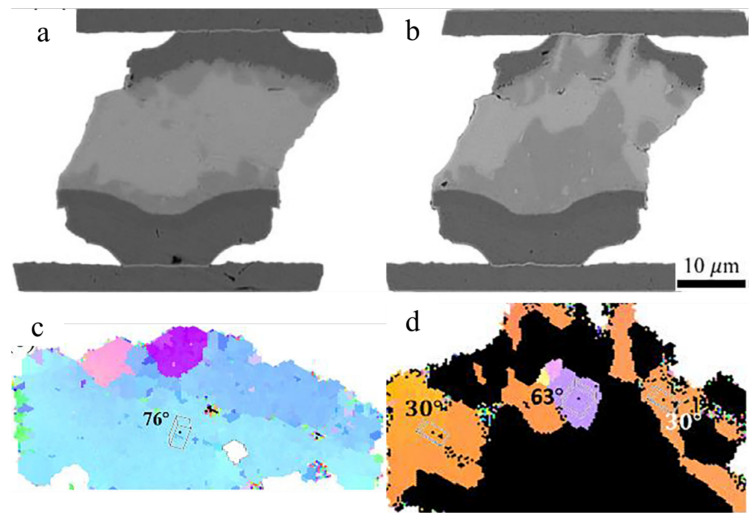
SEM image of Sn-2.5Ag solder microbumps with (**a**) high α-angle grains and (**b**) low α-angle grains. (**c**,**d**) The Sn orientation maps of (**a**) and (**b**), respectively. Reprinted with permission from ref. [89]. Copyright 2018 Elsevier.

**Figure 10:**
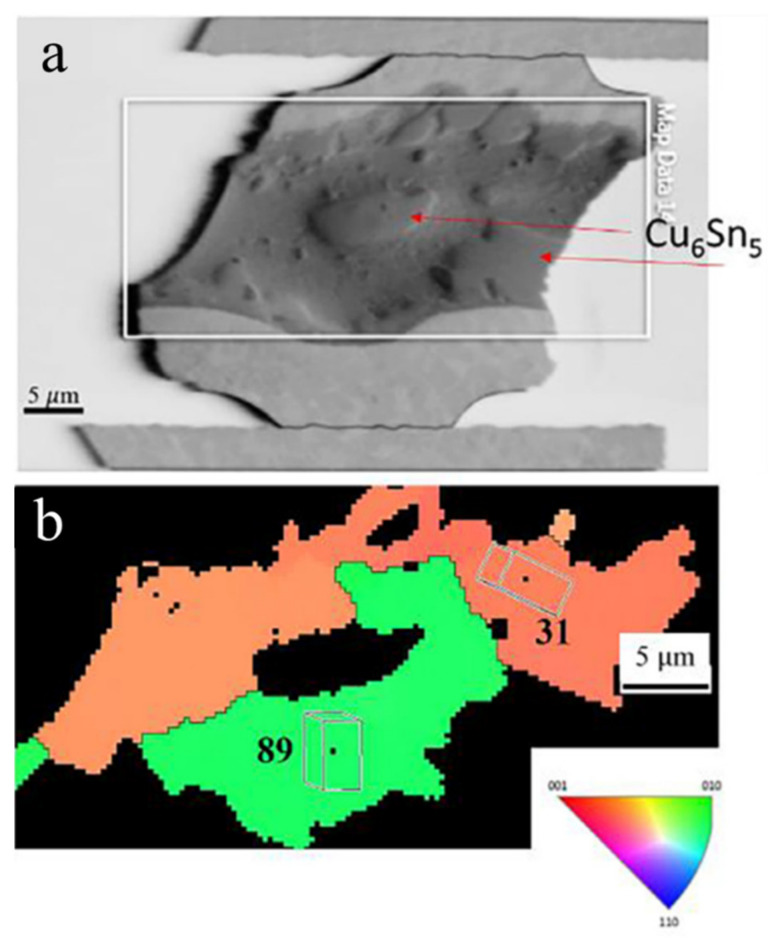
Cu-Sn IMC formation along a grain boundary between 31° and 89° α-angle grains: (**a**) SEM image; (**b**) orientation image map. Reprinted with permission from ref. [89]. Copyright 2018 Elsevier.

Another study reported the effect of the Sn grain orientation on Cu-Sn IMC growth during thermomigration in microbumps [89]. The chip with Cu/Sn2.3Ag/Ni microbumps was examined between a heat source at 200 °C and a heat sink at 100 °C for 150 h. Considering that the Ni of the UBM was at the cold end, the Cu_6_Sn_5_ formation was caused by the Cu flux from the Cu UBM at the hot end during thermomigration. When the α-angle was high (76°), the UBM dissolution was very low (Figure 9a,c). On the contrary, the considerable IMC growth at the cold end and serious UBM dissolution at the hot end were obvious in a microbump with low α-angle grains (Figure 9b,d). Additionally, the Cu-Sn IMC formation in the solder of the microbump is of interest, as shown in Figure 10a. According to the EBSD results, the massive Cu flux was blocked by a grain boundary (Figure 10). The IMC was formed on a grain boundary between the grains with α-angles of 31° and 89°, as shown in Figure 10a. Due to the Sn c-axis in the top grain near the hot end being parallel to the thermogradient, large amounts of Cu migrated via the low α-angle grain to the cold end. Then, Cu migrations were blocked by the high α-angle grain near the cold end. Therefore, the IMC formation in the cold-end region was not occupied by high α-angle grains, as can be observed in Figure 10b. A similar phenomenon was also observed during electromigration [90]. Additionally, only in the 150 h TM test were the asymmetrical IMC formation and UBM dissolution along low α-angle grains much more significant in solder joints with a low bump height (~15 μm) than that with a bump height of 280 μm for the 600 h TM-test.

The results included a higher thermogradient in lower bump height solder joints under the same test conditions. In other words, the effect of the Sn grain orientation on the TM degradation is significant owing to the serious thermogradient and few dominant grains.

## 4. Effect of Sn Grain Orientation on Thermomechanical Fatigue

**Figure 11:**
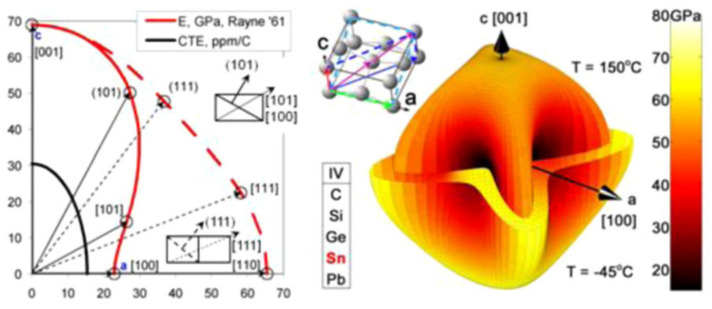
The distribution of the CTEs and the elastic modulus in the Sn crystal. Reprinted with permission from ref. [99]. Copyright 2011 Springer Nature.

**Figure 12:**
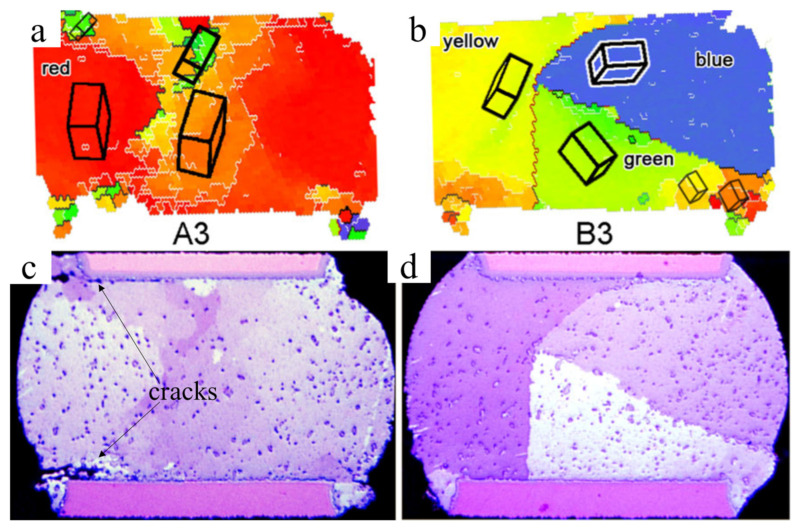
(**a**) Sn crystal orientation map of (**c**) a solder joint with crack propagation after TCT. (**b**) Sn crystal orientation map of (**d**) a solder joint without crack propagation. The black boxes show the Sn crystal unit cells in the solders. Reprinted with permission from ref. [99]. Copyright 2011 Springer Nature.

The CTE mismatch between the polymer substrate (PCB, interposer) and the die packaging (wafer-level packaging, packaging-on-packaging, and 3DIC chip sets) induces TMF damage in solder joints during temperature cycling tests (TCTs) [30,91,92,93,94,95]. In some studies, recrystallization via thermal strain induced TMF cracks in high-strain regions during TCTs, and crack propagation occurred inside the solder in joints close to the interface regions [30,96,97,98]. Moreover, Thomas et al., reported on the influence of the Sn grain orientation on the TMF behavior in Sn-Ag-Cu solder joints when the occurrence of cracks appeared much faster in some joints than in other joints in a chip–solder joint–board assembly [97]. This phenomenon is caused by the anisotropic Young’s modulus and CTE of Sn. Figure 11 shows that the CTE and Young’s modulus along the c-axis are 2–3 times larger than those along the a-axis [39,99]. Clearly, there are different levels of thermal stress and strain, resulting in different Sn grain orientations during the TCTs. The CTEs of the Si, c-axis, and a-axis in Sn are 3, ~30, and 15 ppm/°C, respectively. The largest mismatch of CTEs occurred between the Si and Sn solders with Sn grains of the c-axis parallel to the interface [97]. This phenomenon can be demonstrated via simulations [100,101]. Additionally, the slip system of Sn is {110}/[001]. When the c-axis of Sn is parallel to the interface, it is also parallel to the shear stress direction, causing dislocation slips to occur more easily, resulting in crack propagation at the interface with high thermal stress concentrations (Figure 12a) [99]. Conversely, cracks do not occur in solders when the Sn grains of the c-axis are not parallel to the interface (Figure 12b). As shown by the simulation results, the angle between the c-axis and the normal direction of the board surface significantly affected the inelastic strain energy density at the interface between the solder and the UBM over one temperature cycle. The highest inelastic strain energy density occurred in the solder whose c-axis was almost parallel to the normal direction of the board surface [100]. The order of the degrees of recrystallization (the damage accumulation) for angles of 0°, 45°, and 90° of the c-axis was identified via EBSD. Herein, this failure induced by the Sn grain orientations is defined as mode-Ⅰ.

**Figure 13:**
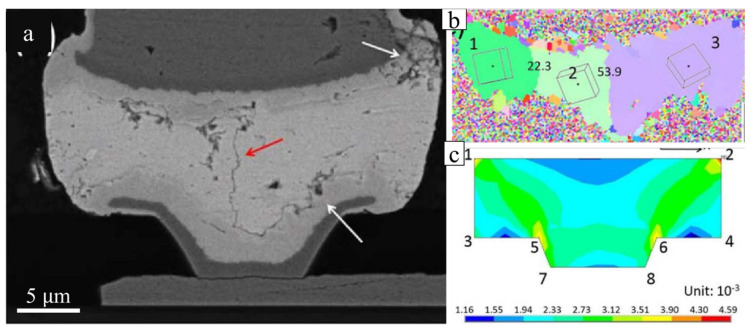
(**a**) SEM image of a microbump with crack propagation (pointed out by the red arrow) along the grain boundary with high misorientation (53.9°) near the corner with high thermal strain after the TCT. (**b**) EBSD orientation map (the boxes show the Sn crystal unit cells in the solders, and the numbers are the misorientations of the boundaries) and (**c**) thermal strain distribution shown via finite-element method simulation in the microbump of (**a**). Reprinted with permission from ref. [102]. Copyright 2017 Springer Nature.

**Figure 14:**
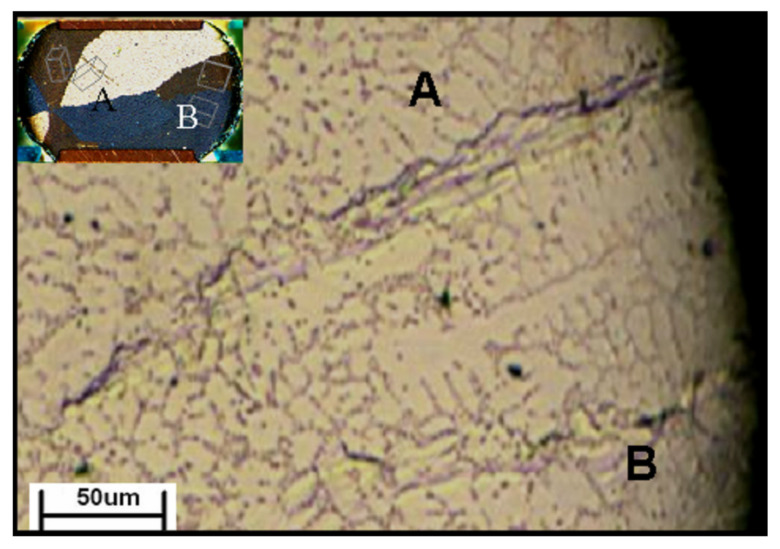
Crack propagation along the grain boundary between grain A and B in a solder joint between two polymer substrates. Reprinted with permission from ref. [29]. Copyright 2007 Elsevier.

Compared to the chip–polymer substrate assembly, solder joints between the chip–chip assemblies in stacking packages (PoP and 3DIC) are widely seen, and the thermal stress from CTE mismatch is rare because the chips are single-crystal Si. However, the CTE mismatch would be caused by the CTE differences in Sn grains as c- and a-axes. In this failure mode, the cracks will be observed along the grain boundaries in the solders, but not at the interface between the solder and substrates [102]. As the results in Figure 13a show, a crack, highlighted by the red arrow, propagated across the solder. From the EBSD analysis in Figure 13b, the crack was along a grain boundary with a misorientation of 53.9°. On the other hand, cracks were not observed along the grain boundary with a misorientation of 22.3°. The CTE mismatch between the c- and a-axes showed a 2-fold difference, which should induce crack formation along the grain boundaries, especially those with high misorientations. If the misorientation of a boundary between two Sn grains is low, the thermal strain would be limited due to the non-influential CTE mismatch. The cracks at the corners, highlighted by white arrows, were likely caused by the shape of the microbump rather than mode-Ⅰ because the c-axis in grains is almost parallel to the interface. Figure 13c shows that critical strains occurred at the corners of points 1, 2, 5, and 6, where the cracks were located. Additionally, the crack propagation along grain boundaries can also be seen in the polymer–polymer substrate assembly (Figure 14) [29], proving that the origin of the CTE mismatch was from the solder rather than from the substrates. In Figure 14, the Sn grain boundary is a kind of cyclic twin boundary that shows resistance against the mechanical strain and stress. Unfortunately, after TCTs, the crack propagation induced by the CTE mismatch between the Sn grains was much more effective than that resisted by the CTBs. This failure is defined as mode-Ⅱ. Therefore, after combining mode-Ⅰ and mode-Ⅱ, the recommended Sn grain to avoid thermal strain failure is a single-crystal structure where the Sn c-axis is not vertical to the interface.

## 5. Method of Sn Grain-Orientation Control in Solder Joint

**Figure 15:**
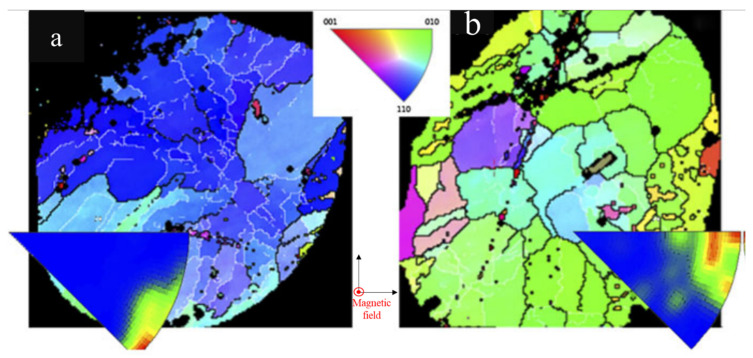
The EBSD orientation maps of two solder joints, both with the Sn c-axis vertical to the magnetic field. The Sn (**a**) [110] and (**b**) [010] orientations are parallel to the magnetic field. Reprinted with permission from ref. [105]. Copyright 2015 Springer Nature.

As mentioned in Section 2, Section 3 and Section 4, we understand that the Sn grain orientation plays a very important role in Sn-rich solder joint reliability, influencing the EM, TM, and TMF. Controlling the Sn grain orientation during the solder solidification of the reflow process is worth investigating. In this review, we suggest two methods to control the Sn grain orientation in solder joints. The first one is used to fabricate a solder joint in a magnetic field during the solidification. In some studies, the crystal orientation was highly related to the direction of the magnetic field during solidification [103,104]. Chen et al., found that the Sn c-axis preferred to be vertical to the *y*-axis, which is close to the electron flow during electromigration, as shown in Figure 15. Figure 15a,b show the Sn’s preferred orientations of [110] and [010] to the normal direction in solder joints, respectively [105]. Although the magnetic field cannot completely control the grain orientation in the normal direction, the c-axis is significantly vertical to the direction of the magnetic field. They suggested that this is caused by the Sn’s anisotropic magnetic susceptibility, which is controlled by a constant to evaluate a material’s magnetization within an imposed magnetic field.

The free energy change of the system during solidification can be described as:

(11) $$\Delta G=\Delta G_{0}+\Delta E$$

where $\Delta G_{0}$ and ΔE are the changes in free energy without and with the magnetic field, respectively; ΔE is proportional to the magnetic susceptibility of the material [103]. In the study, the magnetic induction intensity levels of Sn c- and a-axes under the magnetic field intensity level of 50,000 are −0.00297 and −0.002775, respectively. The magnetic susceptibilities of the Sn c- and a-axes are 1.77 × 10^−6^ and 8 × 10^−6^, respectively. Therefore, when the magnetic susceptibility is low, the free energy change in the system under constant intensity of the magnetic field is low. As the Sn undergoes solidification, due to the Sn c-axis having a smaller magnetic susceptibility than the a-axis, the direction of solidification would be aligned to the direction with the lowest free energy, inducing the c-axis to become vertical to the magnetic field. Moreover, the difference in magnetic susceptibility between the Sn c-axis and a-axis is dependent on the intensity of the magnetic field, i.e., an increase in the intensity of the magnetic field may result in a more significant preferred orientation of the Sn grain [103]. The magnetic field controlling the grain orientation of Sn can be realized using induction heating (IH) technology with magnetic field applications [106,107].

**Figure 16:**
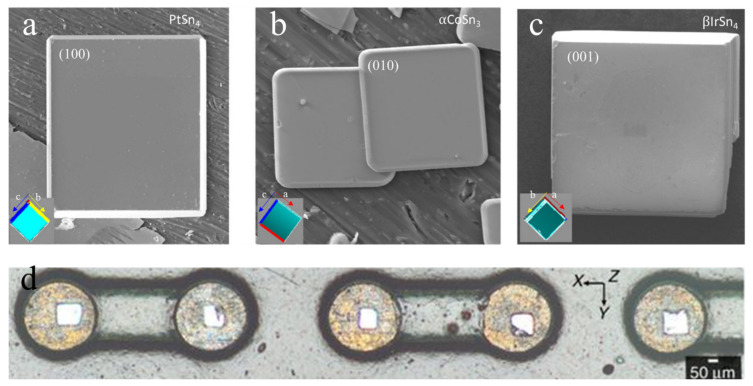
The orientation control of the seed crystals of (**a**) PtSn_4_, (**b**) αCoSn_3_, and (**c**) βIrSn_4_ bonded to Cu pads (**d**). Reprinted with permission from ref. [108]. Copyright 2017 Springer Nature.

**Figure 17:**
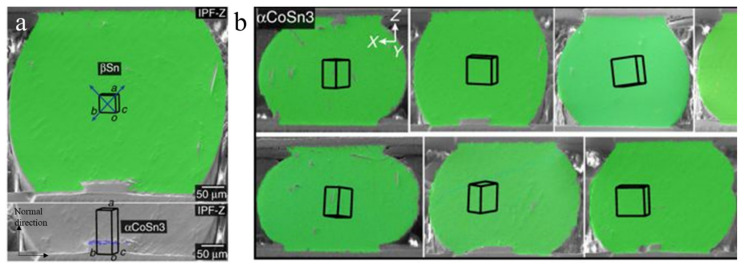
(**a**) The a-axis of Sn controlled by the a-axis of the αCoSn_3_ seed crystal during the solidification of Sn-rich solder joints. (**b**) The solder joints of the Sn single crystal with controlled Sn grain orientations during the reflow process (the boxes show the Sn crystal unit cells in the solders). Reprinted with permission from ref. [108]. Copyright 2017 Springer Nature.

The other method involves the control of the Sn grain orientation using seed crystals of single-crystal IMCs of PtSn_4_, αCoSn_3_, and βIrSn_4_ [108,109]. The characteristics in common for these IMCs are the body-centered tetragonal (BCT) lattice and good lattice match to the Sn. The long axes of PtSn_4_ (Figure 16a), αCoSn_3_ (Figure 16b), and βIrSn_4_ (Figure 16c) are the a-, b-, and c-axes, respectively. When the IMC seed crystals were bonded to Cu pads via transient liquid phase bonding (Figure 16d), they controlled the long axes directed to the normal direction (Figure 17a). Then, Sn-3.5Ag solder balls with the Sn a-axis along the long axes could be achieved during a reflow process, achieving control of the Sn grain orientation in the solder joints, as shown in the Cu/Sn-3.5Ag/αCoSn_3_/Cu solder joints in Figure 17b. This also could be achieved by PtSn_4_ and βIrSn_4_. In addition to the control of the Sn grain orientation, single-crystal Sn solder was fabricated using the seed crystals, the use of which is important to avoid crack propagation along the grain boundaries during TCTs in Sn-rich solder joints.

## 6. Summary

The Sn grain orientation plays an important role in affecting the failure mode during EM, TM, and TMF in Sn-rich solder joints.

In EM, Sn grains with low α-angles accelerate the IMC formation at the anode during EM due to the rapid interstitial diffusion along the c-axis of Sn. Although void formation at the cathode along the Sn grains with high α-angles is observed in flip–chip solder joints, it plays no role in the line-type solder joint that eliminates the current crowding effect on the EM. The UBM dissolution and IMC formation that occur along the c-axis rather than the electron flow prove the domination of the Sn grain orientation on the EM. When the solder size is miniaturized to a microbump, the short diffusion length can amplify the early failures caused by Sn grains with low α-angles, inducing considerable IMC growth at the anode and serious UBM dissolution at the cathode. The anisotropic diffusions caused by the Sn grain orientation during EM were also observed in TM studies.

On the other hand, without CTBs in Sn-rich solders, IMC growth occurs along the Sn grain boundaries with high misorientations instead of along the Sn lattices. During TCTs, the crack propagation at the interface between the solder and UBM in flip–chip solder joints is called the TMF of mode-I in this study. The TMF of mode-II is defined as crack propagation along the grain boundaries. The former is caused by the large CTE mismatch between the Si and Sn grains with the c-axis parallel being rather than that with the a-axis oriented to the FR4 board, while the latter is caused by the CTE mismatch between Sn grains with substantially anisotropic differences. The former is rarely observed in the solder joints between substrates of the same material.

Moreover, the anisotropic magnetic susceptibility of Sn results in the c-axis aligning vertically to the magnetic field during Sn solidification and seed crystals with a BCT structure and a good lattice match to the Sn (PtSn_4_, αCoSn_3_, βIrSn_4_), which not only controls the Sn grain orientation, but also results in single-crystal Sn solders during the solidification of the Cu/SnAg/Cu solder joints.

## Figures and Tables

**Table 1:** The diffusivity levels of Cu and Ni calculated at 120 °C.

Type of Diffusion	Diffusivity (cm^2^/s)	Condition	Ref.
D_Cu in Sn_	5.96 × 10^−6^	Along c-axis	[36]
	9.7 × 10^−8^	Along a-axis	[36]
D_Ni in Sn_	8.22 × 10^−5^	Along c-axis	[37]
	1.10 × 10^−9^	Along a-axis	[37]
